# Nursing Leadership in Practice: A Qualitative Scoping Review of Framework Implementation Experiences in Public Healthcare Systems

**DOI:** 10.1155/jonm/8807131

**Published:** 2026-03-21

**Authors:** Luke Marks, Jessica Biles, Rachel Kornhaber

**Affiliations:** ^1^ School of Nursing, Paramedicine and Healthcare Sciences, Faculty of Science, Charles Sturt University, Bathurst, New South Wales, Australia, csu.edu.au; ^2^ Quality Clinical Safety and Nursing Directorate, Western NSW Local Health District, Orange, New South Wales, Australia; ^3^ School of Dentistry, Faculty of Medicine and Health, The University of Sydney, Sydney, New South Wales, Australia, sydney.edu.au; ^4^ School of Health, University of New England, Armidale, New South Wales, Australia, une.edu

**Keywords:** advanced practice, evidence-based practice, implementation, leadership frameworks, nursing leadership, workplace culture

## Abstract

**Aim:**

To explore how nurse leaders in public healthcare systems experience the implementation of professional practice and leadership frameworks and to identify contextual factors that influence their practical application.

**Background:**

Leadership frameworks are widely promoted to enhance nursing leadership capability, yet their translation into practice remains inconsistent. Understanding how nurse leaders experience these frameworks is essential to inform leadership development and organisational strategy.

**Methods:**

A qualitative scoping review was conducted using Braun and Clarke’s reflexive thematic analysis. Fourteen qualitative studies published between 2015 and 2024 were identified through systematic searches of CINAHL, Scopus and MEDLINE. The review followed the Arksey and O’Malley framework and PRISMA‐ScR guidelines.

**Results:**

Five themes were identified: leadership skills, leadership development, leadership challenges, evidence‐based practice and workplace culture. Leadership was described as a responsive practice shaped by clinical credibility, strategic management and interprofessional influence. A conceptual model, the Clinical–Administrative–Research Nexus, emerged reflecting the concurrent demands placed on nurse leaders. Systemic barriers such as hierarchical constraints, limited resources and inconsistent organisational support contributed to a persistent ‘knowing‐doing’ gap. Workplace culture was the most influential factor, shaping leadership development, evidence translation and team cohesion.

**Conclusion:**

Leadership frameworks were experienced as contextual tools rather than standalone solutions. Their implementation depends on structural support, mentorship and alignment with clinical realities. Leadership success was shaped more by the conditions in which it is enacted than by individual capability alone.

**Implications for Nursing Management:**

The findings highlight the need for integrated system‐level support to enable nurse leaders to navigate the complexities of their clinical, administrative and research responsibilities. Organisational strategies must move beyond individual skill building to address structural barriers and foster cultures that support leadership development and evidence‐based practice.

## 1. Introduction

Healthcare systems face growing pressure from ageing populations, workforce shortages and care complexity [1, 2]. These pressures are especially acute in nursing. The World Health Organization [3] reports a global nursing workforce shortage of 5.8 million nurses as of 2023, with projections indicating a reduction to 4.1 million by 2030 through coordinated international efforts. In Australia, a deficit of over 70,000 nurses is anticipated by 2035 [4], while the USA expects a shortfall of 63,000 registered nurses by 2030 [3].

Rapid career progression, driven by attrition and skill shortages, has placed nurses into leadership roles with limited preparation. This ‘jumping up the ladder’ contributes to stress, burnout and role confusion [5]. Nurse leaders operate across clinical, administrative and interprofessional domains, often with inadequate support or training [6, 7]. While resource constraints and hierarchical barriers are common, cultural factors also shape leadership effectiveness [8].

Nursing leadership is increasingly recognised as essential to healthcare transformation. Evidence links effective leadership to improved patient safety, care quality and organisational performance [9]. Systematic reviews have demonstrated the impact of transformational and relational leadership styles, which emphasise staff empowerment, shared decision‐making and relational trust, and have been associated with reduced patient mortality, fewer adverse events and improved nurse job satisfaction [10–14].

Despite this, a persistent gap remains between nursing leadership theory and practice. Many healthcare organisations struggle to implement and sustain leadership frameworks that support nurse leaders in real‐world settings [15]. Two types of frameworks are commonly used: professional practice frameworks and models, which guide clinical governance, care standards and professional accountability [16], and leadership‐focused frameworks and competency models, which emphasise strategic capabilities such as decision‐making and team management [7, 17]. While both types of frameworks aim to enhance nursing practice, professional frameworks align with clinical and regulatory standards, whereas leadership frameworks focus on cultivating leadership behaviours.

However, the translation of frameworks into everyday practice is poorly understood, particularly in relation to whether they adequately prepare leaders for the simultaneous demands of real‐world healthcare settings [18]. Leaders often implement these frameworks without sufficient experience or mentorship, especially in contexts affected by rapid staff turnover [5].

The COVID‐19 pandemic further highlighted the importance of nursing leadership. Nurse leaders played pivotal roles in crisis response and workforce management, yet many lacked the structural support needed to sustain these efforts [19]. These challenges underscore the importance of understanding how leadership frameworks are experienced and enacted in practice.

Therefore, this scoping review synthesises qualitative research on nurse leaders’ experiences implementing professional practice and leadership frameworks in public healthcare systems internationally. By focusing on qualitative methodologies, it aims to identify contextual factors, barriers and enablers that influence the practical application of these frameworks and inform future leadership development strategies.

In this review, leadership refers to formal or designated nursing roles that involve responsibility for influencing practice, supporting staff development, guiding clinical or organisational decision‐making, or implementing professional practice and/or leadership frameworks. Accordingly, the population includes management roles (e.g., nurse managers and unit managers) and advanced practice or education‐based roles with embedded leadership responsibilities (e.g., nurse practitioners, clinical nurse specialists, clinical nurse consultants and clinical nurse educators). This operational definition is author‐defined and informed by established nursing leadership and professional practice literature, which characterises leadership as involving clinical influence, organisational responsibility and systems‐level decision‐making [7, 16, 17].

## 2. Methods

### 2.1. Aim

This scoping review aims to explore how nurse leaders experience the implementation of professional practice and leadership frameworks in public healthcare systems, and to identify factors that facilitate or inhibit their practical application.

### 2.2. Research Question

How do nurses in advanced practice and management roles experience the implementation of professional practice and leadership frameworks within international public healthcare systems?

### 2.3. Design

This scoping review examined primary qualitative research to explore evidence on factors that facilitate or impede nursing leaders’ implementation of professional practice and leadership frameworks. The methodology was guided by the Arksey and O’Malley [20] framework, which includes five key stages: (1) identifying the research question, (2) identifying relevant studies, (3) study selection, (4) charting the data and (5) collating, summarising and reporting the results. The review process adhered to the Preferred Reporting Items for Systematic Reviews and Meta‐Analysis (PRISMA) extension for scoping reviews [21]. A completed PRISMA‐SCR checklist is provided as a supporting file (available here).

### 2.4. Search Methods

The PCC framework (Population, Concept, Context) (see Table 1) recommended by the Joanna Briggs Institute guided this scoping review. The Population included nursing leaders or educators with a leadership role, such as nurse practitioners, clinical nurse consultants, clinical nurse specialists, nurse managers and clinical nurse educators. Leadership roles were determined using the definitions and descriptions provided within each included study, relying on job titles and stated responsibilities where available, and authors’ descriptions of leadership or education functions where titles varied across contexts. The Concept included leadership dynamics, framework implementation and factors influencing success, such as enablers and barriers. The Context was limited to public healthcare systems internationally. These parameters informed the eligibility criteria (see Table 1) and the search strategy (see Table 2).

**TABLE 1:** Inclusion and exclusion criteria used for study selection.

	Inclusion	Exclusion
Population	Nursing leaders or educators in leadership roles, including nurse practitioners, clinical nurse specialists, advanced practice nurses, clinical nurse consultants, nurse managers/unit managers and clinical nurse educators.	Other healthcare or nonhealthcare professionals; nurses and midwives not in leadership or education roles.
Concept	Implementation of leadership frameworks or strategies; interventions involving organisational change to support leadership development; use of tools or technologies to support leadership frameworks; impact of frameworks on leadership skills, effectiveness and professional development.	Interventions unrelated to leadership frameworks; general management theories without clinical relevance; theoretical frameworks without applied evidence; unit‐based initiatives; short professional development courses not embedded in broader frameworks; outcomes unrelated to leadership or nursing practice.
Context	Public healthcare settings (e.g., wards, facilities, Local Health Districts); peer‐reviewed, English‐language, qualitative primary research.	Private practice settings; non‐nursing professions; studies outside public healthcare systems; grey literature, reviews, discussion papers, editorials; mixed‐methods or quantitative studies.

**TABLE 2:** Scopus search strategy applied during the literature review.

((“nurse practitioner^∗^” OR “clinical nurse specialist^∗^” OR “advanced practice nurse^∗^” OR “physician assistant^∗^” OR “clinical nurse consultant^∗^” OR “nurse manager^∗^” OR “Nurse administrator^∗^” OR “nurse executive^∗^” OR “clinical nurse educator^∗^” OR “nurse unit manager^∗^”) OR (nurs^∗^ N3 lead^∗^) OR ((MH “Nursing Leaders+”) OR (MH “Advanced Practice Registered Nurses”) OR (MH “Clinical Nurse Specialists+”) OR (MH “Nurse Practitioners+”) OR (MH “Clinical Nurse Leaders”) OR (MH “Nurse Executives+”)) AND ((MH “Leadership”) OR (“clinical leadership” OR “leadership theor^∗^” OR “leadership strateg^∗^” OR “leadership model^∗^” OR “leadership principle^∗^”)) AND ((MH “Conceptual Framework”) OR (MH “Professional Practice”) OR (MH “Nursing Practice”) OR (MH “Nursing Practice, Evidence‐Based”) OR leadership N4 competenc^∗^) or (“professional practice” OR “leadership framework^∗^” OR “conceptual framework^∗^”) OR (MH “Professional Standards”) OR (MH “Practice Improvement”) OR “evaluation framework^∗^” OR “quality improvement project^∗^”) AND ((MH “Program Implementation”) OR TI(implement^∗^ OR develop^∗^ OR application^∗^ OR governance^∗^ OR guidance))

*Note:* “∗” represents a Boolean wildcard used in the Scopus search strategy to capture multiple word endings.

**FIGURE 1:**
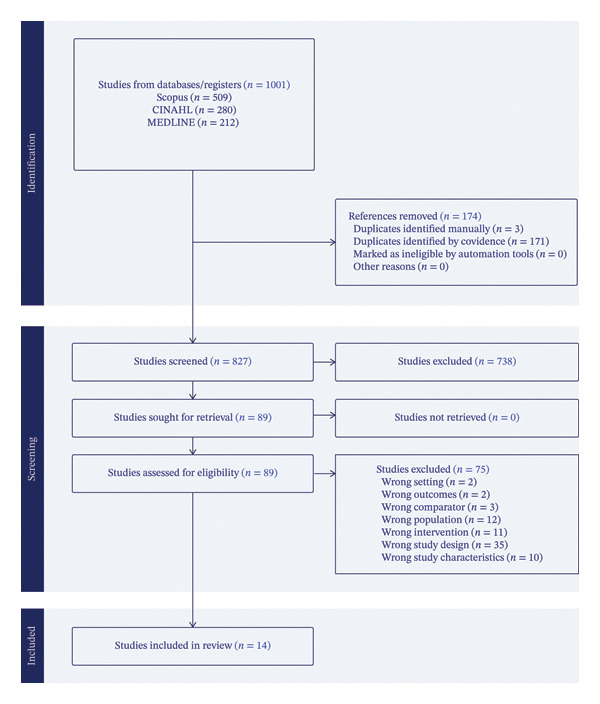
PRISMA flow diagram illustrating the study selection process (adapted from Ref. [21]).

In November 2024, a comprehensive literature search was conducted using Cumulative Index to Nursing & Allied Health Literature (CINAHL) via EBSCOhost, Scopus and MEDLINE (via Ovid, including MeSH headings from 1996 to present). The Boolean operators AND/OR were used with targeted subject headings and keywords. Only articles published between January 2015 and November 2024 were included, reflecting a period of increased research linking transformational leadership to improved outcomes [9, 11]. The Scopus search strategy is detailed in Table 2, and the PRISMA diagram in presented in Figure 1.

### 2.5. Inclusion and Exclusion Criteria

Studies were included if they were peer‐reviewed, English‐language qualitative research focused on nursing leaders in advanced practice or management roles, as defined in the PCC framework (see Section 2.4 and Table 1). Screening decisions were guided by pre‐established criteria aligned with the PCC framework. The full inclusion and exclusion criteria are summarised in Table 1.

### 2.6. Search Outcome

Articles were downloaded using EndNote and imported into Covidence, which removed duplicate entries. A total of 827 records were screened. Title and abstract screening and full‐text review were conducted independently by two researchers (Luke Marks and Jessica Biles). Disagreements were resolved through discussion with a third researcher (Rachel Kornhaber). Fourteen studies met the inclusion criteria and were included in the final review (Figure 1).

### 2.7. Quality Appraisal

Although formal critical appraisal is not required for scoping reviews, this study adopted a trustworthiness approach using a ‘code‐then‐converge’ strategy [22]. All three researchers independently coded the data and then met to compare, challenge and validate emerging themes. This process enhanced the credibility of the findings.

### 2.8. Data Abstraction

Data were extracted by the lead author (Luke Marks) and organised into a table of study characteristics, including author, year, country, aim, participants, study design, data collection and analysis methods, framework or tool implemented and key findings (see Table 3). Participant roles were extracted and reported as described by the primary study authors. A separate column was included to capture the type of leadership training reported in each study, with a note that these were not considered frameworks unless they met criteria for structured implementation.

**TABLE 3:** Summary of included studies with key characteristics and findings.

Author, year, country	Aims	Participant	Study design	Data collection and analysis	Type of leadership training	Key findings
Cheng et al. [23], China	To explore leadership of Chinese nurse managers in evidence‐based nursing implementation	*n* = 15 nurse managers. Average age 41 years; average nursing experience 22 years; average management experience 10 years	Qualitative secondary data	Purposive and theoretical sampling; semistructured interviews; directed content analysis	Kouzes & Posner’s Five Practices of Exemplary Leadership® model	Five leadership practices identified: (1) Modelling the way, (2) Inspiring a shared vision, (3) Challenging the process, (4) Enabling others to act, (5) Encouraging the heart two Implementation phases: (1) Getting oneself prepared, (2) Keep it going
Chen et al. [24], China	To explore the prospective acceptability of a prototype for an implementation leadership training programme that was codeveloped with nurse managers in Hunan province, China	*n* = 14 nurse managers from three tertiary hospitals; all F; median age 42 years (range 32–48); median nursing experience 23 years; median management experience 12 years (range 3–19); 13 held master’s degrees	Qualitative descriptive	Purposive sampling, semistructured interviews, thematic analysis	Ottawa Model of Implementation Leadership; Five‐module leadership training programme with coaching and group work	Seven main themes: (1) Affective attitude, (2) Burden, (3) Ethicality, (4) Intervention coherence, (5) Opportunity costs, (6) Perceived effectiveness, (7) Self‐efficacy Programme showed high acceptability for developing leadership competencies
Feo et al. [25], Australia	To report the process and outcomes of codesigning a nursing leadership program for fundamental care	Stage 1: *n* = 60 workshop participants from 11 countries, *n* = 40 survey respondents. Stage 2: *n* = 19 from nine countries	Participatory action research with two‐stage codesign process	Stage 1: Online workshops, qualitative content analysis. Stage 2: 3‐day face‐to‐face workshop with interactive presentations and group discussions	Fundamentals of Care Leadership Program (codesigned intervention)	Four main themes: (1) Advocacy/lobbying for fundamental care, (2) Understanding fundamental care concepts, (3) Implementation and measurement strategies, (4) Building supportive networks
Fleiszer et al. [26], Canada	To describe how actions of nursing unit leaders influenced the long‐term sustainability of a best practice guidelines (BPG) program on inpatient units	*n* = 39 participants from four nursing units in large urban academic health centre; 14 organisational informants, 25‐unit informants including nurse managers, educators and clinical nurses	Qualitative descriptive case study	Semistructured interviews, content analysis	Best Practice Guidelines Program (falls prevention, pressure ulcer prevention, pain management)	Four main themes: (1) Unit‐level leadership impact extending beyond management to mentoring and enabling, (2) Integrated implementation approach with aligned vision and strategy, (3) Professional practice development focused on building collective capacity, (4) Communication mechanisms including group handovers and face‐to‐face forums
Harvey et al. [27], Australia	To explore how different nursing and midwifery leadership roles enact responsibility for implementing evidence‐based practice	*n* = 14 nursing and midwifery leaders from 1 local health network and two metropolitan hospitals; included senior leaders, unit managers, consultants, practitioners and educators across organisational levels	Qualitative descriptive case study	Purposive sampling, semistructured interviews, content analysis	Two leadership approaches: top‐down/managerial and bottom‐up facilitative	Three main themes: (1) Knowledge sources varied by leadership approach, (2) Implementation strategies differed between managerial and facilitative approaches, (3) Implementation challenges were consistent across leadership styles
Hughes et al. [28], Malta	To understand how the participants ascribed meaning to their experiences of developing as leaders within the Maltese context.	*n* = 6 nurse leaders in advanced practice, administration and academia; 83% F; mean age 46 years; median leadership experience 12 years	Phenomenological	Purposive sampling, semistructured interviews using field notes, open and axial coding	Authentic Leadership Framework (Avolio and Gardner, 2005): Self‐awareness, internal moral perspective, balanced processing, relational transparency	Three main themes: (1) Cultural integration within Maltese healthcare context, (2) Developing as a leader through experience and reflection, (3) Self‐reflection as essential component of authentic leadership
Kjerholt and Hølge‐Hazelton [29], Denmark	To describe whether an action learning‐inspired journal club for nurse leaders can develop the leaders’ self‐perceived competences to support a research culture in clinical nursing practice.	*n* = 4 nursing leaders; three held master’s degrees; varying qualification and leadership experience levels	Participatory approach‐action learning‐based, pilot study using a multimethod approach	Field observations, facilitator logs, group interviews, email correspondence, meeting minutes; manifest and latent content analysis	Action learning‐inspired Journal Club for Nurse Leaders	Three main themes: (1) Safe peer‐based learning environment valued by participants, (2) Enhanced awareness of research culture development and improved leadership confidence, (3) Implementation challenges included workload barriers to reflection between meetings
Miltner et al. [30], USA	To investigate the relevance of planned programmatic change to local nurse leaders.	*n* = 20 nurse managers in focus groups (demographics from *n* = 33 total); mean age 45.1 years; mean nursing experience 20.1 years; 82% F; 70% white; 67% bachelor’s degree	Qualitative	Three concurrent focus groups, content analysis	American Organisation of Nurse Executives and American Association of Critical‐Care Nurses, Nurse Manager Leadership Partnership framework: the science, the art, the leader within	Three main themes: (1) Managing versus leading role conflicts, (2) Gaining a voice in organisational decision‐making, (3) Garnering support from administration and peers
Pereira et al. [31], Brazil.	To understand the meaning of transformational leadership (TL) from the perspective of nursing managers in an emergency and intensive care network and use the journal club strategy to develop a learning environment for the exercise of TL.	*n* = 9F emergency and intensive care managers (seven completed both phases); average age 38 years; management experience 3–20 years	Qualitative action research	Convenience sampling; semi‐structured interviews; Strengths, Weaknesses, Opportunities and Threats (SWOT) matrix analysis; content analysis	Journal club strategy combined with SWOT matrix planning tool; transformational leadership theory	Three main themes: (1) Team‐related factors, (2) Leadership process factors, (3) Leader role factors. Journal club and planning model provided effective praxis for leadership development
Prestia [32], USA	To explore how Chief Nursing Officers (CNOs) sustain themselves in the practice of nurse executive leadership in an acute care medical centre and to update findings in the literature.	*n* = 20 CNOs from acute care centres; 18F, 2M; mean age 55.7 years; mean RN experience 32.8 years; mean CNO tenure 5.1 years; all minimum 2+ years in the current role	Interpretative phenomenology	Semistructured interviews, qualitative analysis	Ray’s theory of bureaucratic caring, authentic leadership theory, resiliency theory	Six main themes: (1) Loving the profession, (2) Having broader impact, (3) Reflecting on one’s work, (4) Learning to manage conflict, (5) Maintaining work/life balance, (6) Working with supportive leaders
Rumsey et al. [33], South Pacific	To strengthen nursing and midwifery leadership and capacity in developing countries in the Pacific.	*n* = 34 nurses and midwives from 12 South Pacific countries; 89%F; age range 28–57 years; all held nursing/midwifery roles and qualifications	Qualitative descriptive study (part of a larger health system strengthening program)	Semistructured interviews; inductive thematic analysis	Australian Award Fellowship program: 14‐day leadership program with lectures, group work, project planning, mentorship and 18‐month follow‐up	Four main themes: (1) Having country‐wide objective for change, (2) Learning how to be a leader through structured program, (3) Negotiating implementation barriers, (4) Having effective mentorship support
Wang et al. [34], China	To elucidate strategies for improving organisational adaptation during public health emergencies from the perspectives of nurse managers.	*n* = 17 nurse managers from various settings; 16F, 1M; age range 29–52 years; average experience 22 years (range 6–32); settings included hospitals, health centres, shelters and communities	Qualitative	Purposive sampling; semistructured interviews with field notes; Interpretative Phenomenological Analysis framework incorporating Colaizzi’s method	Complex Adaptive Systems Theory examining nonlinear dynamism and adaptability during emergencies	Three main themes: (1) Seeking institutional support for environmental adaptation (identity, information, training, supplies, planning, family care), (2) Building intrateam support for structural adaptation (goals, management, relationships, environment), (3) Activating individual capacity for behavioural adaptation (role modelling, personnel matching, needs satisfaction, recognition)
Xu et al. [35], China	To develop a leadership and management competency framework applicable to Chinese nurse champions guided by the competency matrix for clinical nurse leader (CNL).	*n* = 27 clinical nurse managers from six tertiary hospitals in Shanghai; average age 45.8 years; experience levels: six (< 5 years), 10 (5–10 years), 11 (> 10 years); 11 ward head nurses, 16 department head nurses	Qualitative	Semistructured interviews with field notes; deductive and inductive content analysis	CNL competency matrix (2016) from Commission on Nurse Certification	Three main themes: (1) Nursing leadership (horizontal leadership, advocacy, care integration, collaboration, clinical expertise), (2) Clinical outcomes management (quality improvement, knowledge management, evidence‐based practice), (3) Care environment management (finance, systems, policy, informatics, law, emergency response)
Ylimäki et al. [36], Finland	To describe advanced practice nurses (APN)s’ experiences of EBP implementation in the Finnish hospital setting.	*n* = 12 APNs from two university hospitals; 10F, 2M; average age 47.5 years; education: three master’s applied science, seven master’s nursing science, two nursing science doctorates; average APN experience 3.25 years (range 1–7.5)	Qualitative descriptive	Purposive sampling, thematic interviews, inductive content analysis	FinAME (Finnish Action Model of Expertise) for strengthening EBP implementation and clarifying expert roles	Four main themes: (1) Evidence‐Based Practice (EBP) in clinical nursing practice, (2) EBP leadership roles and responsibilities, (3) Implementation support mechanisms, (4) EBP integration in APNs’ daily work

*Note:* F: female; M: male.

### 2.9. Thematic Analysis Approach

Braun and Clarke’s [37] six‐step reflexive thematic analysis was used. This method supports the synthesis across diverse qualitative designs without reliance on a single theoretical framework [37] (p. 78). The process included familiarisation with the data, semantic coding, pattern identification, theme development and refinement. A reflexive approach was maintained throughout, with attention to researcher assumptions and the diversity of international contexts [37]. The lead researcher (Luke Marks) identified five primary themes.

## 3. Results

### 3.1. Study Characteristics

Fourteen qualitative studies published between 2015 and 2024 were included, with half published in the last five years. Studies were conducted across diverse regions, including China (*n* = 4), Australia (*n* = 2), the USA (*n* = 2) and one each from Finland, Malta, Denmark, Brazil, Canada and the South Pacific. Participants included registered nurses (*n* = 20), advanced practice nurses (*n* = 44) and nurse managers (*n* = 124), with most holding postgraduate qualifications. Participant role labels are presented as reported in the included studies and should be interpreted as descriptive categories; they may overlap (e.g., some nurse managers are also registered nurses). Methodologies varied, including qualitative descriptive designs, phenomenology, participatory approaches and secondary data analysis, offering a rich foundation for thematic synthesis. Paper characteristics are provided in Table 3.

### 3.2. Thematic Findings

**FIGURE 2:**
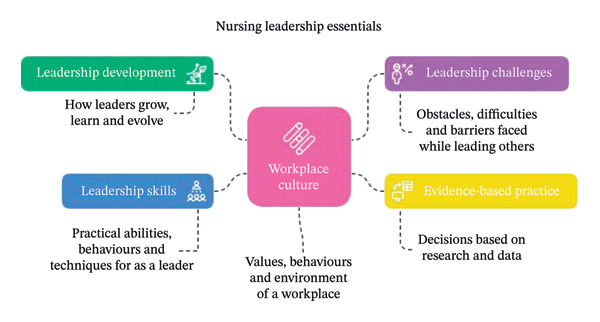
Thematic map of five identified themes representing nursing leadership essentials (image generated using Napkin AI).

Five major themes emerged from the thematic analysis of 14 articles: *Leadership Skills*, *Leadership Development*, *Leadership Challenges*, *Evidence-Based Practice* and *Workplace Culture*. These themes revealed complex interconnections between leadership capabilities, development pathways and organisational contexts (see Figure 2).

#### 3.2.1. Theme 1: Leadership Skills

This theme involved four subthemes. Leadership skills were described as dynamic and contextually shaped, encompassing strategic management, communication, interprofessional collaboration, emotional resilience and empowerment [23, 25, 27–32, 34, 35]. These skills were not viewed as innate traits but as practices developed through experience, reflection and responsiveness to organisational and cultural contexts. Although emotional resilience and cultural context were not designated as seperate subthemes, they emerged consistently across the data and are discussed further in Themes 2 and 5.

##### 3.2.1.1. Strategic Management

Leaders described strategic management as requiring persistence and an iterative approach [26, 32, 34]. During the COVID‐19 pandemic, one manager explained, ‘*…redefined the workflow in one night…’* [34] (p. 7). Leadership required sustained engagement and responsiveness to both team and organisational dynamics. Central to this was persistence: ‘*Kept at it…eventually [the nursing staff] got it and did it*…’ [26] (p. 314). As one leader explained, ‘*It has to be alive!’* [26] (p. 314).

##### 3.2.1.2. Communication

Communication was a leadership skill requiring adaptability and audience awareness [30, 32]. One leader explained, ‘*…to your staff is in this fashion, and then to physicians is in this fashion…’* [30] (p. 255), reflecting situation awareness. Assertiveness was also valued: ‘*I don’t mince words…’* [32] (p. 579), but always with a focus on patient outcomes: ‘*…move [communication] roadblocks by keeping patients central…’* [32] (p. 579).

##### 3.2.1.3. Interprofessional Collaboration

Interprofessional collaboration required clinical credibility and assertiveness, with leaders advocating across disciplines and influencing beyond nursing [25, 35]. One participant noted, ‘*…must be a good clinician…otherwise why would others listen…’* [35] (p. 968). Leaders advocated across disciplines: ‘*…remind doctors, nurses and other professionals to pay attention…’* [35] (p. 966) and recognised the need to influence beyond nursing: ‘…*leaders need to have the ability to influence and gain support from other leaders outside of nursing’* [25] (p. 7).

##### 3.2.1.4. Empowerment

Empowerment strategies included mentorship, recognition and shared authority to foster team growth and protect staff well‐being [23, 24, 27, 31]. One leader described, ‘*I noticed a staff nurse eager to learn…So I decided to provide her with more chance to “show off”…’* [23] (p. 674). Structured training supported leadership development [24], while recognition was expressed through public and verbal acknowledgement: ‘*…putting their names on the education materials…’* [23] (p. 676) and ‘*I thank them and then say: “that was a difficult shift, but you guys killed it”’ ’*[31] (p. 4).

#### 3.2.2. Theme 2: Leadership Development

Leadership development was a prominent theme, reflecting how nursing leaders grow through formal education, identity formation, self‐development and mentorship [23–25, 27–29, 31, 33, 35]. Leadership development involved four subthemes.

##### 3.2.2.1. Formal Education

Formal education was consistently described as foundational to leadership development [23, 24, 35]. One participant explained: ‘*A leader must learn leadership theory and use it, so that they can lead teams scientifically rather than just by work experience’* [35] (p. 965). This highlighted the perceived limitations of experience‐only learning and the value placed on structured preparation. Preferences for comprehensive training were also evident, with one participant describing it as involving ‘*…various activities such as lectures, experience sharing, group discussions, presentations, plan development and implementation’* [24] (p. 3725). These reflections suggest that leadership education must be both theoretical and practical.

Leaders also described the need to bridge knowledge gaps through self‐directed learning. One participant reflected, ‘*I realized my deficit of the knowledge…Then I read articles and attend workshops so that I would be capable to locate the evidence…’* [23] (p. 676). Another emphasised the importance of context‐specific application: ‘*I will be more interested if…the training program will focus on finding solutions and make plans for solving my clinical problems’* [24] (p. 3725).

Specialised knowledge was seen as essential to leadership practice, particularly in areas beyond clinical expertise. Participants described the need for economic awareness, systems navigation and digital literacy to support effective decision‐making [35]. These insights reflect the expanding scope of leadership roles and the need for cross‐functional competence.

##### 3.2.2.2. Professional Identity Development

Professional identity was a key driver of leadership motivation and resilience [32, 33]. Leaders expressed pride and belonging: ‘*I am a nurse. I could never see myself as anything else’*, and ‘*I don’t tell people I am a nurse executive…I tell them I am a nurse’* [32] (p. 578). This identity anchored their leadership practice in clinical values and relational care. One participant noted: ‘*I am proud to be a nurse, and pride sustains my commitment’* [32] (p. 578).

Leadership identity evolved through exposure to new environments. One participant reflected: ‘*…I have learnt a lot to be courageous, to be confident and to be networking, to be collaborating’* [33] (p. 55). Leadership was increasingly viewed as a practice of influence, as one leader explained: ‘*When I 1st got out of nursing school, I had a 2 patient assignment…now every day my practice allows for the safe and effective practice of nursing…’* [32] (p. 579).

##### 3.2.2.3. Self‐Development

Self‐development was described as a continuous, experiential process shaped by informal learning, reflection and emotional regulation [27, 29, 31, 32]. Participants reflected on the absence of formal instruction, noting that leadership growth often occurred through practice. One leader explained: ‘*I suppose I’ve learnt as I’ve gone along…No-one’s shown me how to do it’* [27] (p. 61), while another added, ‘*I learn every day…what you learn anecdotally and practically is very valuable’* [27] (p. 60). Reflective spaces were considered essential, with journal clubs described as ‘*…like a kind of free period, and we leaders need that’* [29] (p. 46)*.* Resilience was also built through learning from failure: ‘*Sometimes we are going to make a decision* that is *not the best…we learn from it’* [32] (p. 579). Emotional regulation supported recovery and sustainability, with leaders describing routines such as ‘*My 45 min drive home is my time to think…’ and ‘…I build time for myself so that I can recharge…’* [32] (p. 579). In high‐pressure environments, emotional control was necessary: ‘*…be less emotional…look more pragmatically’* [28] (p. 416).

##### 3.2.2.4. Mentorship and Support

Mentorship and professional networks were critical to leadership development [25, 28, 29, 33]. One leader explained: ‘*Leaders need mentors, I learned a lot from mentors…reflection with people whom I trust’* [28] (p. 416), highlighting the importance of relational learning. Exposure to diverse leadership styles was encouraged: ‘*Get multiple opinions, ask questions, seek [your] enemies—they are your best asset…’* [28] (p. 416), suggesting that leadership is shaped through observation and dialogue.

Networks provided motivation and connection, particularly through international engagement and peer collaboration. Safe learning environments were critical: ‘*…in a small group with my peers, where I don’t have to keep up a facade because I’m a leader…’* [29] (p. 46). Mentorship was described as ongoing and collaborative: ‘*Yes I feel I have all the support and she [mentor]…has played a big part in planning this thing…’* [33] (p. 56).

#### 3.2.3. Theme 3: Leadership Challenges

Leadership challenges involved four subthemes, encompassing knowledge implementation difficulties [24, 36], psychological pressures [30, 36], hierarchical constraints [27, 28] and resource limitations [30, 33]. These challenges shaped the leadership experience across diverse healthcare contexts, often creating tension between aspiration and reality.

##### 3.2.3.1. Knowledge Implementation

Difficulties in applying evidence to practice were a consistent challenge across the studies [24, 36]. Leaders expressed uncertainty about their role in facilitating change, with one manager stating, ‘*…I still don’t understand how these leadership behaviours influence point-of-care nurses implementing EBP’* [24] (p. 3728). The complexity of leadership models further contributed to confusion: ‘*I heard about the three types of behaviours…but I don’t know how I can use them together…’* [24] (p. 3728). Despite awareness of best practice, implementation remained inconsistent, as staff reported ‘*…variability and inconsistency in nursing care’* [36] (p. 229).

Access barriers compounded these challenges. Leaders described difficulty locating guidelines: ‘*People do not read guidelines, they cannot find them…not easily accessible in practice…’* [36] (p. 231). The dominance of medical sources created further gaps: ‘*…the guidelines are current care guidelines, which are more medical related’* [36] (p. 230). In the absence of nursing‐specific resources, staff often relied on external expertise: ‘*When we look at where we get our evidence from, generally is from our [medical] consultants’* [27] (p. 59).

##### 3.2.3.2. Psychological Pressures

The emotional burden of leadership was evident across multiple studies [30, 36]. Leaders described feeling isolated when implementing change: ‘*Many times it feels as if you are all alone in these issues…’* [36] (p. 230). One manager described the emotional toll of leadership, noting, ‘*…you have to get so mean…to exist as a manager…’* [30] (p. 255), while another questioned the sustainability of excessive workloads: ‘*Working 60 or 80 h a week…am I really productive, though?’* [30] (p. 254).

##### 3.2.3.3. Hierarchical Constraints

Hierarchical and cultural norms were identified as barriers to leadership effectiveness [27, 28]. In traditional environments, resistance to change was deeply embedded. One leader explained, ‘*Sameness is part of Maltese culture…’* and ‘*…this culture still needs to be told what to do time and time again’* [28] (p. 415). Educational structures remained static, with participants noting, ‘*The curriculum for nursing has not changed in 10 years. Everyone thought it was excellent because it was brought from Liverpool’* [28] (p. 412). This comment reflects a broader cultural resistance to change, where tradition is valued over innovation. Such inertia limits the integration of contemporary leadership content, leaving emerging nurse leaders underprepared for the complexities of modern healthcare. These reflections suggest that leadership efforts must contend not only with organisational inertia but also with cultural expectations that reinforce outdated practices.

##### 3.2.3.4. Resource Limitations

Resource constraints were a persistent barrier to leadership practice across multiple domains [24, 30, 33]. Staffing shortages were particularly acute: ‘*We are short of nursing staff now, and nurses are already overloaded with daily work…’* [24] (p. 3728), making it difficult to introduce new practices that ‘*…would need more training and extra energy to integrate into nursing practice…’* [24] (p. 3728). Time constraints affected all aspects of leadership, with managers reporting, ‘*…we don’t have the time for the dialogue…or…go to a class’* and ‘*you’ve got to look at the budget…’* [30] (p. 255).

Operational barriers extended to basic needs. One manager explained, ‘*[the] process…always gets shut down. It can be anything from obtaining supplies to manpower to you-name-it…’* [30] (p. 255). Professional development was limited, particularly in resource‐constrained settings. One nurse described the lack of support: ‘*…no one coming in to upgrade our knowledge or help improve – no updates with what is happening with other countries…they are just there [to] work, work, work’* [33] (p. 55). These reflections reveal tensions between leadership aspirations and system realities, where structural constraints undermine innovation.

#### 3.2.4. Theme 4: Evidence‐Based Practice

Evidence‐based practice reflects the evolving relationship between research knowledge and clinical implementation [23, 24, 26, 27, 35, 36]. This theme is explored through three subthemes.

##### 3.2.4.1. Evidence‐Based Practice

Leaders described a growing conceptual awareness of EBP, making a shift from tradition‐based care to research‐informed decision‐making [24, 36]. This transition was accompanied by uncertainty. One participant reflected, ‘*In the past, our nursing practices were more based on the previous experiences; now the idea of evidence-based practice is gradually being accepted’* [24] (p. 3725), while another admitted, ‘*I don’t know what exactly should I do’* [24] (p. 3725). Despite this, the perceived value of EBP was clear: ‘*I understand the importance of EBP implementation for clinical nursing…I believe it is well-needed to learn the evidence-based ways to solve problems in my unit’* [24] (p. 3729). Confidence in clinical decisions was enhanced when supported by research: ‘*I will be more confident to make the decision when I know it is supported by previous research’* [24] (p. 3725).

Despite increased awareness, as reflected in the comment that ‘*…people’s awareness has increased…’* [36] (p. 229), leaders acknowledged variability in understanding. This inconsistency, ‘*…*w*ho understands how deeply the meaning of the term varies…’* [36] (p. 229), posed additional challenges for leadership practice.

##### 3.2.4.2. Research Application

Applying research in practice required more than awareness. It demanded methodological competence and contextual sensitivity [23, 35]. Leaders described the role of nurse champions who ‘*…search high quality literature and extract key information…so that everyone can be convinced…’* [35] (p. 968), while also recognising the complexity of implementation, ‘*Applying evidence to patient care is complex…we also need to consider patient preferences and costs…’* [35] (p. 968).

Methodological literacy was seen as foundational; ‘*Method will help nurses know what was behind the evidence. Nurses can use the same method to solve future problems’* [23] (p. 676). Leaders who possessed both knowledge and research confidence could challenge established practices: *‘…*“ *dry wound healing” was the predominant treatment…I did [moist wound healing]. The results came out good…’* [23] (p. 674).

However, knowledge did not always translate into practice. One participant observed that, *‘Nursing staff also has knowledge on how something should be done, although then it is not always completed in this way on a practical level…’* [36] (p. 229). Uncertainty about leadership influence further complicated implementation.

##### 3.2.4.3. Implementation Methods

Organisations adopted diverse strategies to support EBP, including structured quality improvement models and educational initiatives [24, 26, 27, 35, 36]. Formal methodologies such as ‘*…Plan-do-check-act circle (PDCA), 6σ, quality control circle (QCC)…’* [35] (p. 968) provided frameworks for evidence integration. Systematic review processes ensured currency: ‘*…everything that’s on our policies and procedures and guidelines portal is up to date because those procedures and policies and guidelines are reviewed frequently’* [27] (p. 59).

Training programs aimed to build evidence‐seeking capacity through structured educational and collaborative learning opportunities [24]. Network‐based dissemination strategies were also employed: ‘*…this kind of network of contact people whom we educate…’* [36] (p. 231). Some leaders pursued integrated approaches, ‘*trying to link all the different approaches: BPG, elderly friendly, patient engagement, environmental safety, therapeutic conversations…by implementing a new method of rounds…’* [26] (p. 315).

Despite these efforts, engagement remained limited. One organisation noted that, ‘*…we have those research clubs…but participation in these is very minimal’* [36] (p. 230). Experiences with training programs created scepticism: ‘*…they (the trainers) left after the training, and no one cared about how our plans [developed in the training] were implemented’* [24] (p. 3728). These reflections underscored the importance of sustained support and follow through in EBP initiatives.

#### 3.2.5. Theme 5: Workplace Culture

Workplace culture emerged as a foundational theme that intersects with and influences all aspects of nursing leadership practice. It encompasses the environmental, structural and normative factors that shape how leaders operate, how evidence‐based practice is implemented, how teams function and how professional development occurs [24, 27, 28, 31, 33–36]. The findings revealed three subthemes.

##### 3.2.5.1. Cultural Norms and Professional Dynamics

Cultural norms shaped leadership agility and implementation success, with organisational size and relational dynamics influencing responsiveness and team cohesion [28, 31]. In smaller organisations, leaders described enhanced accessibility: ‘*…small so you can talk to the person in charge…’* [28] (p. 412), enabling rapid implementation of leadership initiatives. In Brazil, relational norms prioritised respect and unity: ‘*…respect is fundamental’* [31] (p. 4), demonstrating how cultural context influenced leadership style and team dynamics.

Traditional hierarchies continued to shape evidence‐seeking behaviours, with guidance often ‘*…generally…from our [medical] consultants’* [27] (p. 59), reflecting embedded power structures that limited nursing autonomy. However, cultural evolution was evident in some contexts, ‘*…the hierarchy is changing, so nurses are more assertive’* [28] (p. 412), suggesting a shift toward more collaborative professional relationships.

Competitive dynamics undermined leadership development as one participant noted, ‘*If you are selected to be in the program instead of other people they will put in complaints’* [33] (p. 55). In contrast, cultures that emphasised collective progress supported leadership growth: ‘*…the way we…lead our colleagues and understanding the values, behaviours, and norms of where we come from…knowing that we can change the behaviours and…values’* [33] (p. 55).

Unity and shared purpose were seen as essential: ‘*We all need to speak the same language, the team needs to be on the same page’* [31] (p. 3). Yet, role ambiguity persisted: ‘*We are quite poorly familiar with our work descriptions…so that the people in our organization also do not know’* [36] (p. 230), creating confusion that impacted both individual and team performance.

##### 3.2.5.2. Organisational Support Systems

Organisational structure significantly influenced leadership effectiveness and innovation capacity. Smaller organisations demonstrated agility and accessibility, enabling rapid implementation of leadership initiatives and EBP [28]. Gender dynamics shaped team culture, with one leader reflecting *‘…in a female-dominated team, we shared a sense of self-esteem and group honour. We wanted to let others know that “we can make it even in hard situations…’”* [34] (p. 5). This collective identity strengthened resilience and motivation: ‘*…eager to volunteer for frontline work’* [34] (p. 5), demonstrating how cultural identity supported challenging implementations.

Organisational commitment to EBP required systematic support. Leaders emphasised that, *‘…education and the support absolutely I think is what is fundamental. If it’s not on the agenda of the organisation, it ain’t going to happen…’* [27] (p. 60). However, inconsistent middle management support created barriers: *‘If the [Unit Manager] doesn’t believe…“you’re giving me too much work and we’re too busy doing something”, it doesn’t happen…’* [27] (p. 60), highlighting how individual leaders could shape or obstruct cultural progress.

Collaborative structures were valued: ‘*Collaborative partners are also very important…’* [36] (p. 230), yet structural gaps persisted: ‘*We, in fact, at least do not have such structures with clinically specialized nurses, that we would collaborate together’* [36] (p. 230), indicating missed opportunities for professional integration.

##### 3.2.5.3. Environmental Creation and Sustainability

Leadership approaches that prioritised positive workplace atmospheres were consistently valued. One participant stated, ‘*A leader should be able to create a good working environment…full of positive energy…so that everyone will like to stay’* [35] (p. 967), linking environment creation to staff retention and engagement. Proactive communication fostered psychological safety: ‘*…encouraged our nurses to voice any discomfort…As a result, this created a positive motivation for nurses…to return to their positions once they had recovered…’* [34] (p. 11). These strategies supported individual well‐being and organisational resilience.

Resource constraints threatened cultural sustainability, with professional development often deprioritised: ‘*…development is then like the first* that is *eliminated, if there is no time for something’* [36] (p. 231), undermining long‐term leadership capacity. Platform‐based support systems offered promise, as one participant noted, ‘*people (managers) will see this training program as a long-term program and take it more serious’* [24] (p. 3728), and in addition, it then ‘*provides an easier way to get support and resources’* [24] (p. 3728). Sustained organisational commitment and infrastructure are critical to overcoming resource‐related cultural barriers and enabling leadership development.

## 4. Discussion

**FIGURE 3:**
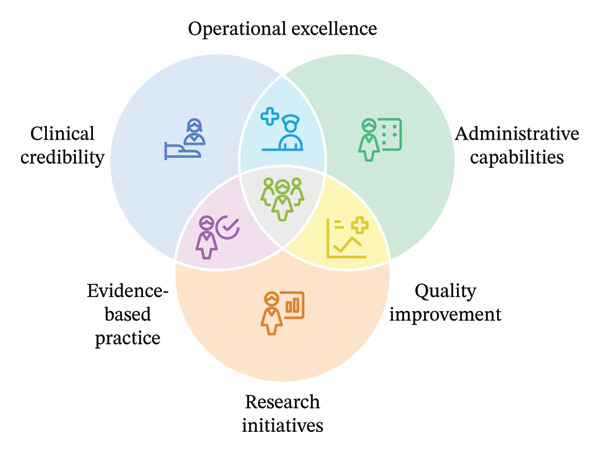
Clinical–Administrative–Research Nexus framework: Venn diagram synthesizing leadership domains from the reviewed studies (image generated using Napkin AI based on author analysis).

This review aimed to explore how nurse leaders experience the implementation of professional practice and leadership frameworks in public healthcare systems. Through the process of analysis and interpretation, the proposed model has been conceptualised as the Clinical–Administrative–Research Nexus framework (Figure 3), reflecting nursing leadership as a profession‐specific model requiring clinical credibility, administrative capability and research utilisation. Research literacy is recognised as a core component of leadership development. The Nexus framework reflects the complexity of leadership roles in public healthcare systems and extends existing leadership models by recognising the concurrent demands placed on nurse leaders [14, 15, 38, 39]. Clinical credibility was consistently positioned as foundational to interprofessional respect and influence [6, 17].

Despite sustained investment in leadership development, nursing leaders continue to face implementation challenges [40]. Ahmadi and Vogel [41] describe this as a ‘knowing‐doing gap’, where leaders understand theoretical concepts but struggle to apply them due to systemic constraints. Similar patterns are evident in studies by Hughes [42] and Välimäki et al. [12] although they do not use this terminology. These findings suggest that knowledge alone is insufficient without structural support for practice.

EBP was consistently recognised as a core leadership capability across the included studies [23, 24, 27, 35, 36], aligning with broader literature that positions EBP as essential to contemporary nursing leadership [43]. While Currey et al. [44] proposed the Clinical Nurse Research Consultant role to bridge clinical and research domains, this model does not fully address the administrative pressures faced by nurse leaders [45, 46].

In some settings, medical hierarchies continue to influence nursing autonomy and decision‐making [47–49], prompting nurse leaders to adopt strategic approaches tailored to interprofessional dynamics. The term ‘leadership squeeze’, describes the simultaneous pressures of clinical, administrative and interprofessional responsibilities that constrain leadership capacity [8, 12]. Conventional training programs often fail to address these structural challenges, which stem from organisational systems rather than individual shortcomings [12].

Persistent barriers despite capability‐building efforts indicate that boosting individual skills alone cannot overcome systemic limitations [43, 50]. Workplace culture significantly shapes leadership effectiveness [51–53], reinforcing the need for context‐sensitive approaches. While transformational leadership remains influential, it must be critically adapted to nursing contexts where clinical credibility and evidence translation are central to leadership effectiveness [8, 14, 54].

The current evidence base lacks sufficient representation from rural or remote settings [12, 55, 56], limiting understanding of how the Nexus framework functions in resource‐constrained environments. Rural leaders face unique challenges, including limited access to professional development, increased role complexity and geographical isolation, which remain underexplored [55, 56]. Additionally, the relationship between leadership development investments and measurable outcomes such as patient care quality, staff satisfaction and organisational performance remains unclear [57].

Effective nursing leadership requires a paradigm shift from individual skill building to integrated systems‐level support, as supported by evidence from the *Journal of Nursing Management* [58]. While individual capability remains important [47, 48], it must be cultivated within organisations that support cross‐domain integration. Organisational redesign is needed to enable concurrent engagement in clinical, administrative and research roles, aligning leadership practice with strategic workforce planning and policy reform [44, 59–61]. This includes interprofessional governance, joint academic–clinical appointments [44, 45] and culture change initiatives [62] that support the Nexus framework.

These approaches depart from models that treat clinical and leadership skills as aspirational or sequential. Instead, they recognise that nursing leadership effectiveness depends on simultaneous capability across clinical, administrative and research domains, embedded within supportive organisational systems.

## 5. Limitations

This literature review has several limitations. The geographic concentration of studies in Western healthcare systems may introduce cultural bias and limit the global applicability of the findings. The exclusion of grey literature, dissertations and non‐English publications may have omitted relevant insights. Most included studies were cross‐sectional, limiting insight into leadership development across different time periods. Accordingly, the review could not examine whether the relative emphasis placed on specific leadership capabilities (e.g., resilience and interprofessional collaboration) has shifted over time. The absence of intervention studies limits the conclusions that can be drawn about the effectiveness of specific leadership development approaches. Furthermore, this review did not evaluate the contextual appropriateness of specific leadership styles, which may limit generalisability across diverse nursing environments. The limited use of longitudinal designs restricts understanding of how structural barriers and supports for framework implementation emerge, persist or resolve across different stages of implementation.

## 6. Conclusion

Across the included qualitative studies of public healthcare settings, nursing leadership was experienced as a concurrent set of clinical, administrative and research‐related demands captured in the Clinical–Administrative–Research Nexus framework. Leaders described the need to maintain clinical credibility while navigating operational responsibilities and influencing evidence use in practice.

The review identified a persistent gap between leadership knowledge and implementation that was attributed to contextual constraints reported in the included studies, including time pressure, hierarchical structures and limited resources. These findings suggest that leadership framework implementation is shaped by workplace culture, access to mentorship and organisational support systems, rather than individual capability alone.

Within the scope of the reviewed literature, strengthening framework implementation will likely require integrated, system‐level supports that enable nurse leaders to enact leadership in context, including protected time, consistent managerial backing and structured development opportunities aligned with clinical realities. Future qualitative research, including longitudinal designs and greater representation from rural and remote settings, is needed to examine how these contextual conditions evolve and how they influence leadership practice over time.

## Author Contributions

Conceptualisation: Luke Marks, Jessica Biles and Rachel Kornhaber.

Data curation: Luke Marks.

Formal analysis: Luke Marks.

Investigation: Luke Marks, Jessica Biles and Rachel Kornhaber.

Methodology: Luke Marks, Jessica Biles and Rachel Kornhaber.

Project administration: Luke Marks, Jessica Biles and Rachel Kornhaber.

Supervision: Jessica Biles.

Validation: Luke Marks, Jessica Biles and Rachel Kornhaber.

Visualisation: Luke Marks, Jessica Biles and Rachel Kornhaber.

Writing–original draft: Luke Marks, Jessica Biles and Rachel Kornhaber.

Writing–review and editing: Luke Marks, Jessica Biles and Rachel Kornhaber.

## Funding

This research did not receive any specific grant from funding agencies in the public, commercial or not‐for‐profit sectors.

Open access publishing facilitated by Charles Sturt University, as part of the Wiley ‐ Charles Sturt University agreement via the Council of Australasian University Librarians.

## Ethics Statement

Ethical approval was not required for this scoping review, as it synthesised findings from previously published studies and did not involve human participants or primary data collection.

## Conflicts of Interest

The authors declare no conflicts of interest.

## Supporting Information

Supporting File 1: Preferred Reporting Items for Systematic reviews and Meta‐Analyses extension for Scoping Reviews (PRSIMA‐SCR) checklist.

## Supporting information


**Supporting Information** Additional supporting information can be found online in the Supporting Information section.

## Data Availability

The data that support the findings of this study are available from the corresponding author upon reasonable request.
